# High‐Spin States of Manganese(III) Enable Robust Cold‐Adapted Activity of MnO_2_ Nanozymes

**DOI:** 10.1002/advs.202415477

**Published:** 2024-12-16

**Authors:** Qing Tian, Shuaiqi Huangfu, Ge Kang, Haoyu Wang, Huile Liu, Xuejing Wang, Aipeng Li, Yao Chen, Kelong Fan, Lianbing Zhang

**Affiliations:** ^1^ School of Life Sciences Northwestern Polytechnical University 127 Youyi Road Xi'an 710072 China; ^2^ Xi'an Key Laboratory of C1 Compound Bioconversion Technology School of Chemical Engineering and Technology Xi'an Jiaotong University Xi'an 710049 China; ^3^ CAS Engineering Laboratory for Nanozyme Institute of Biophysics Chinese Academy of Sciences 15 Datun Road Beijing 100101 China

**Keywords:** cold‐adapted enzyme, cryogenic catalysis, manganese oxides, nanozyme, spin state

## Abstract

Developing novel cold‐adapted nanozymes and elucidating their mechanisms of action remains a great challenge. Inspired by natural oxidases that utilize high‐spin and high‐valent metal‐oxygen intermediates to achieve high efficiency at low temperatures, in this study, a series of MnO_x_ nanomaterials with varied valence and spin states are synthesized. The activity assay revealed that the oxygen vacancy‐engineered *ε*‐MnO_2_ nanozyme displayed excellent cold‐adapted oxidase‐like properties, and no observable activity loss is observed in the temperature range of −20 to 45 °C. The superior performance is attributed to the high‐spin Mn(III)–O species coupled with its induced Jahn–Teller effect, which facilitates the dissociation and activation of oxygen at low temperatures. As a proof of concept, an excellent cold‐adapted *δ*‐MnO_2_ nanozyme can be obtained using Mn_3_O_4_ as the precursor by regulating the spin state of Mn(III). Moreover, a novel and effective degradation strategy for corn stalk at low temperature is built based on the robust cold‐adapted oxidase‐like activity of *ε*‐MnO_2_. These results not only provide new insights for the rational design of cold‐adapted nanozymes but also broaden the application of nanozymes in low‐temperature industrial processes.

## Graphical abstract

MnO_2_ with a rigid structure is developed as a robust cold‐adaptive nanozyme by regulating its spin state. It exhibited extremely stable oxidase‐like activity under both psychrophilic and mesophilic condition (−20 to 45 °C).

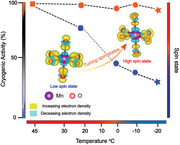

## 1 Introduction

Cold‐adapted enzymes, also called psychrophilic enzymes, are a class of highly temperature‐sensitive enzymes that can effectively catalyze low‐temperature reactions.^[^ ^1^ ^]^ Enabled by the rapid development of biotechnology, cold‐adapted enzymes have been used in food production, pharmaceutical applications, vaccine immunology, environmental remediation, cold chain logistics, fundamental biological research, and other areas.^[^ ^2^ ^]^ Importantly, they can prevent excessive side reactions during thermal catalysis, preserve heat‐sensitive compounds, and provide reliable avenues to reduce energy consumption by allowing the catalysis of chemical reactions that are otherwise difficult to perform at low temperatures.^[^ ^3^ ^]^ However, the loose structure and high flexibility of cold‐adapted enzymes usually result in high sensitivity to metallic ions and organic solvents, along with low variability in thermal stability.^[^ ^4^ ^]^ Furthermore, the lack of enzymatic sources and difficulty in separating and purifying these enzymes greatly restricts the practical application of cold‐adapted enzymes.^[^ ^5^ ^]^ Therefore, the development of alternative cold‐adapted enzymes with high efficiency, good stability, low‐temperature adaptability, and a wide temperature range of operation is of great importance.

Among the various strategies reported for modifying cold‐adapted enzymes or developing alternatives to cold‐adapted enzymes, the use of nanozymes is particular promising.^[^ ^6^ ^]^ Nanozymes, nanomaterials with intrinsic enzyme‐like characteristics, have attracted widespread interest because of their advantages over natural enzymes, including high and tunable catalytic activity, easy batch preparation, and good stability.^[^ ^7^ ^]^ We previously reported the first cold‐adapted nanozyme based on a manganese‐containing metal‐organic framework (MOF), which displays extremely stable oxidase‐like activity in the temperature range of 0–45 °C.^[^ ^8^ ^]^ The catalytic mechanism was proposed to involve a combination of highly active sites, good substrate affinity, and flexible conformation. This mechanism might not be suitable for the design of cold‐adapted nanozymes based on inorganic nanomaterials with rigid structures. Therefore, the development of novel cold‐adapted nanozymes and elucidation of their mechanisms of action remains a great challenge.

In nature, metalloenzymes can activate oxygen at low temperature by forming high‐spin metal‐O species as highly active intermediates,^[^ ^9^ ^]^ providing inspiration for the construction of efficient nanocatalysts at low temperatures. As widely demonstrated, cold‐adapted enzymes feature highly active sites that enable lower reaction activation energies and good substrate affinities at low temperatures to increase product turnover.^[^ ^2^, ^10^ ^]^ Recently, MnO_x_‐based materials have received considerable attention in the field of enzyme mimicking, owing to their multiple valence states coupled with strong oxidizability and diverse crystal structures.^[^ ^11^ ^]^ Although different kinds of MnO_x_ and its derivatives have been fabricated to show multiple enzyme‐like functions,^[^ ^12^ ^]^ no in‐depth exploration of its potential as a cold‐adapted enzyme mimic has been reported. Mn can be easily regulated to exist in different spin states, especially high‐spin Mn(III), rendering them widely applicable in lithium batteries and nanocatalysts.^[^ ^13^ ^]^ Additionally, the electron configuration of Mn ions can be further engineered by numerous strategies, including defects, oxygen vacancies, and valance states, to meet the application requirements.^[^ ^14^ ^]^ Therefore, there is great potential to fabricate MnO_x_‐based cold‐adapted nanozymes by modulating the spin state and structural features for highly active sites and good affinity toward the substrate.

In this study, we fabricated a series of MnO_x_ nanozymes (MnO, Mn_2_O_3_, Mn_3_O_4_, and MnO_2_) with various Mn valence states, morphologies, and crystal phases. Evaluation of their cold‐adaptive properties revealed that *ε*‐MnO_2_ nanozymes presented the most premium cold‐adapted activity with no observable activity loss over a wide temperature range from −20 to 45 °C. Combined computational and experimental approaches have demonstrated that high‐spin Mn(III) improves the dissociation and activation of oxygen atoms by Jahn–Teller (J–T) distortion, thus reducing the reaction energy barrier at low temperatures. Additionally, a novel mild strategy was developed for the catalytic oxidative degradation of corn stalk (CS) at low temperatures. This study provides rational rules for designing cold‐adapted nanozymes based on metal oxide nanozymes without a flexible framework. Innovative progress in low‐temperature catalysts has provided reliable solutions for biomass utilization under mild conditions.

## 2 Results and Discussion

### 2.1 Synthesis, Characterization, and Activity Assay

**Figure 1:**
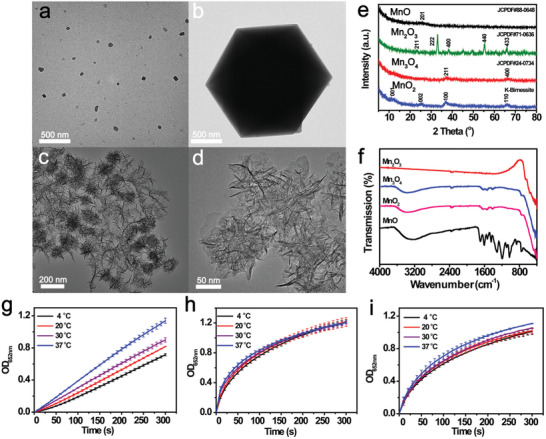
TEM images of a) MnO, b) Mn_2_O_3_, c) Mn_3_O_4,_ and d) MnO_2_ nanoflowers. e) XRD and f) FTIR spectra of the prepared MnO_x_ samples. Kinetic curves of TMB oxidation catalyzed by g) Mn_2_O_3_, h) Mn_3_O_4_, and i) MnO_2_ at different temperatures.

According to our design, a series of MnO_x_, including MnO, Mn_2_O_3_, Mn_3_O_4_, and MnO_2_, were prepared using previously reported methods.^[^ ^15^ ^]^ As indicated by the transmission electron microscopy (TEM) images, the MnO_x_ are different in morphology with spherical nanoparticles, octahedron, and nanoflower covered with petaloid nanoplates identified for MnO (**Figure** **1**a), Mn_2_O_3_ (Figure 1b), Mn_3_O_4_ (Figure 1c), and MnO_2_ (Figure 1d), respectively. Elemental analysis using energy‐dispersive X‐ray spectroscopy (EDS) demonstrated that Mn and O were uniformly dispersed throughout all MnO_x_ samples (Figure S1, Supporting Information). As evaluated by X‐ray diffraction (XRD, Figure 1e) and high‐resolution transmission electron microscopy (HRTEM, Figure S2, Supporting Information) assay, the crystalline patterns of the MnO, Mn_2_O_3_, Mn_3_O_4_, and MnO_2_ were in good agreement with those reported for MnO (JCPDS file: 88‐0648), Mn_2_O_3_ (JCPDS file: 71‐0636), Mn_3_O_4_ (JCPDS file: 24‐0734), and layered birnessite MnO_2_ (JCPDS file: 80–1098), respectively. The presence of Mn–O (519 cm^−1^) and Mn–O–Mn (613 cm^−1^) bonds in all prepared nanozymes were also observed from Fourier‐transform infrared (FTIR) spectra (Figure 1f).

Then, their oxidase‐like activities were assessed at various temperatures using a typical chromogenic reaction with 3,3′,5,5′‐tetramethylbenzidine (TMB) as the substrate. (Figure S3, Supporting Information). As shown in Figure 1g–i, no discernible color change was observed in the MnO‐TMB system. In contrast, the other MnO_x_ samples presented typical oxidase‐mimicking activity with a significant blue color in the system. This implies that the oxidase‐like property might depend on the valence state of the Mn species, and the oxidizing capability of Mn^2+^‐containing MnO was not able to initiate this reaction. As the temperature decreased from 37 to 4 °C, a significant activity depression was observed for the Mn_2_O_3_ (Figure 1g) and Mn_3_O_4_ (Figure 1h). In contrast, MnO_2_ exhibited almost no activity loss in this temperature range (Figure 1i), surpassing both currently reported metal/metal oxide nanozymes and natural enzymes (Figure S4, Supporting Information), which usually exhibits optimum activity at physiological temperatures.

**Figure 2:**
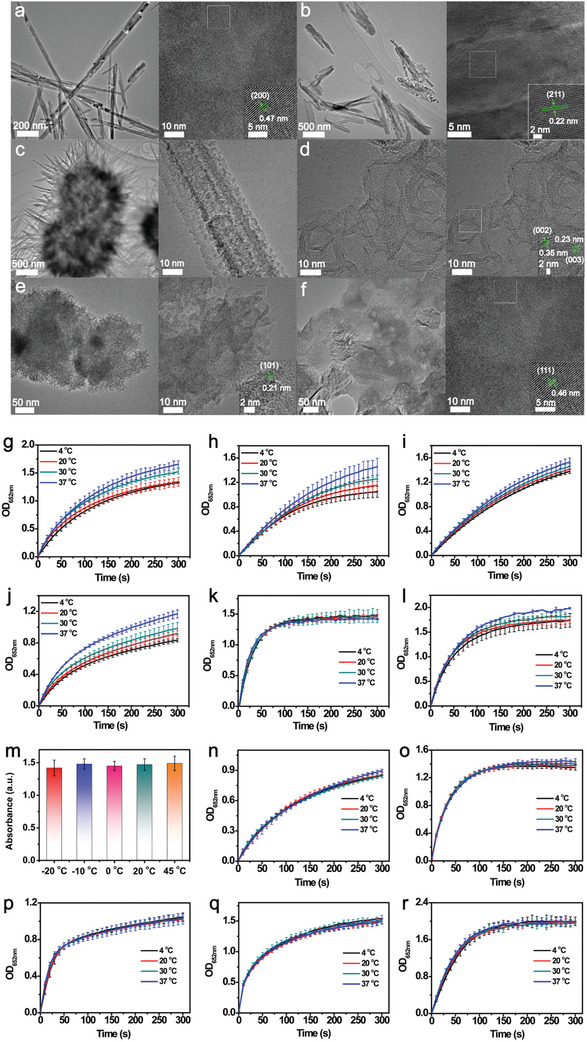
TEM and HRTEM images of a) *α‐*MnO_2_, b) *β*‐MnO_2_, c) *γ*‐MnO_2_, d) *δ*‐MnO_2_, e) *ε*‐MnO_2_, and f) *λ*‐MnO_2_ (inset images represent the HRTEM images of different MnO_2_ samples). Kinetic curves of TMB oxidation catalyzed by g) *α‐*MnO_2_, h) *β*‐MnO_2_, i) *γ*‐MnO_2_, j) *δ*‐MnO_2_, k) *ε*‐MnO_2_, and l) *λ*‐MnO_2_ at different temperatures. m) Optical densities of the *ε*‐MnO_2_–TMB system at 652 nm at different incubation temperatures. Kinetic curves of TMB oxidation catalyzed by n) massive *ε*‐MnO_2_, o) oval‐shaped *ε*‐MnO_2_, p) spherical *ε*‐MnO_2_, q) lamellar *ε*‐MnO_2_, and r) flower‐shaped *ε*‐MnO_2_ at different temperatures.

The inherent characteristics of MnO_2_, encompassing the spin, charge, and orbital states, are often coupled owing to their multiple degrees of freedom and electron correlations.^[^ ^16^ ^]^ Such interactions are highly sensitive to changes in the crystal structure and stoichiometry, and thus likely play an important role in determining cold‐adapted properties.^[^ ^17^ ^]^ Therefore, six kinds of MnO_2_ (*α‐*, *β*‐, *γ*‐, *δ*‐, *ε*‐, and *λ*‐MnO_2_) were fabricated to provide an in‐depth understanding of the relationship between MnO_2_ and its cold‐adapted nanozyme properties.^[^ ^11^ ^]^ The TEM images (**Figure** **2**) and EDS analysis (Figure S5, Supporting Information) clearly revealed the morphologies and high‐resolution structures of the prepared MnO_2_ samples. The HRTEM images (Figure 2a–f, inset images) along with the XRD measurements (Figure S6, Supporting Information), demonstrating that all MnO_2_ samples have crystal structures matching well with reported tetragonal *α*‐MnO_2_ (JCPDS file: 44–0141), tetragonal *β*‐MnO_2_ (JCPDS file: 24–0735), orthorhombic *γ*‐MnO_2_ (JCPDS file: 14–0644), trigonal *δ*‐MnO_2_ (JCPDS file: 80–1098), hexagonal *ε*‐MnO_2_ (JCPDS file: 30–0820), and cubic *λ*‐MnO_2_ (JCPDS file: 44–0992), respectively. Subsequent activity assessments at gradually decreasing temperatures from 37 to 4 °C (Figure 2g–l) revealed that the MnO_2_ samples displayed varied cold‐adapted activities in the order of *ε*‐MnO_2_ > *γ*‐MnO_2_ > *λ*‐MnO_2_ > *α*‐MnO_2_ > *β*‐MnO_2_ > *δ*‐MnO_2_, as induced by the activity variation in the temperature range from 4 to 37 °C. In particular, the *ε*‐MnO_2_ nanozyme exhibited extremely stable activity over a wide temperature range, from −20 to 45 °C, and almost no activity loss was observed (Figure 2m). In addition, we have compared the cold‐adapted activity of *ε*‐MnO_2_ with the previously reported cold‐adapted nanozyme (nMnBTC), and found that *ε*‐MnO_2_ has similar cold‐adapted performance to nMnBTC along with the temperature dropping from 37 to 4 °C, but its catalytic activity is higher than nMnBTC (Figure S7, Supporting Information), confirming the superiority of *ε*‐MnO_2_. It should be noted that the morphology of *ε*‐MnO_2_ has a huge impact on its specific enzyme‐mimicking activity, but no observable influence on its cold‐adapted nanozyme activity (Figure 2n–r; Figures S8, S9, Supporting Information), which excluded the exposed lattice plane as the key factor determining nanozyme activity at low temperatures.

### 2.2 Cold‐Adapted Mechanism of ε‐MnO_2_ Nanozyme

**Figure 3:**
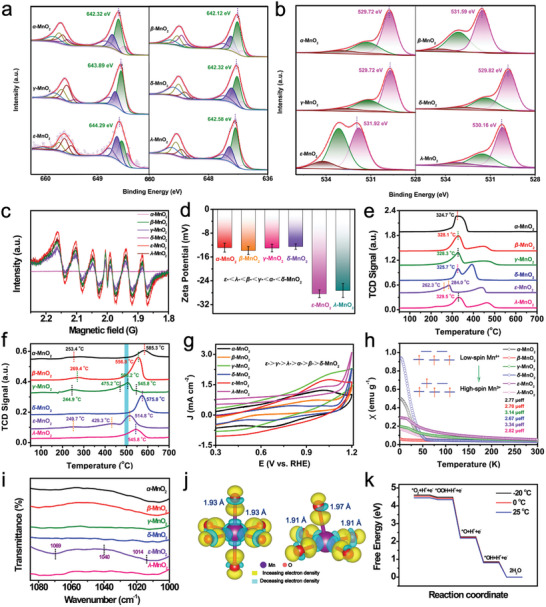
a) Mn 2p and b) O 1s XPS spectra of six types of MnO_2_. c) EPR spectra of MnO_2_–TMB systems. d) Zeta potentials of the six types of MnO_2_. e) H_2_‐TPR profiles; f) O_2_‐TPD profiles; g) CV curves; h) magnetic susceptibility plots; i) Pyridine FTIR spectra of the six types of MnO_2_. j) Calculated differential charge densities of octahedral Mn^4+^O_6_ and Mn^4+^O_5_. k) Energy profile diagram for oxygen reduction reaction on *ε*‐MnO_2_‐OV with TMB as a reducing agent at different temperatures.

Based on these results, in‐depth investigations were conducted to elucidate the catalytic mechanism of *ε*‐MnO_2_. In fact, MnO_2_ is not in a pure Mn^4+^ state, but has a mixed style with the coexistence of Mn^3+^ and Mn^4+^, which suggests that the excellent performance of MnO_2_ might be accounted for by the Mn^3+^ species in the high redox potential with robust oxidizing capability, which is specifically enhanced by Mn^4+^ owing to the induced charge imbalance and structural defects.^[^ ^18^ ^]^ Therefore, the valence states of the Mn species in the MnO2 samples were compared. As illustrated by the Mn 2p X‐ray photoelectron spectroscopy (XPS) data, all MnO_2_ samples were in a mixed state containing Mn^2+^, Mn^3+^, and Mn^4+^ (**Figure** **3**a), except for some differences in the binding energies of the Mn 2p XPS peaks and the average oxidation state (AOS) (Table S1, Supporting Information). It has been reported that Mn(III)–O bonds are longer and weaker than Mn(IV)–O bonds when they coexist in MnO_x_^[^ ^19^ ^]^; thus, a high proportion of Mn^3+^ induces a high oxidation ability. In addition, the O 1s XPS spectrum of MnO_2_ was deconvoluted into lattice oxygen (O_latt_), surface oxygen (O_sur_), and adsorbed oxygen (O_ads_) (Figure 3b). The adsorbed oxygen, rather than the lattice oxygen, was demonstrated to be more favorable for aerobic reactions.^[^ ^20^ ^]^ However, the cold‐adapted activity of MnO_2_ nanozymes was not strongly correlated with the proportion of Mn^3+^, oxygen species, or AOS. Furthermore, the cold‐adapted activity of the MnO_2_ samples was also independent of the number of superoxide radicals (Figure 3c), surface potential (Figure 3d), reducibility (Figure 3e), and oxygen mobility (Figure 3f); thus, the above results do not provide solid evidence to explain the low‐temperature catalytic mechanism of *ε*‐MnO_2_.

Because the redox potential is an important parameter reflecting the oxidation ability of metal oxides,^[^ ^16e^ ^]^ cyclic voltammetry (CV) measurements were conducted on MnO_2_ samples.^[^ ^16d^ ^]^ As shown in Figure 3g, all samples showed typical oxidation peaks in the range of 0.81–1.05 eV, and their oxidation potentials followed a descending sequence of *ε*‐MnO_2_ (1.05 V) > *γ*‐MnO_2_ (1.04 V) > *λ*‐MnO_2_ (1.02 V) > *α‐*MnO_2_ (1.01 V) > *β‐*MnO_2_ (0.96 V) > *δ‐*MnO_2_ (0.81 V), which is correlated positively with their cold‐adapted activity results.

To further elucidate the electron spin configuration of MnO_2_,^[^ ^21^ ^]^ we measured the zero‐field‐cooled temperature dependent magnetic susceptibility (*χ*_m_) (Figure 3h). The total effective magnetic moment (*µ*_eff_) and number of unpaired *d* electrons (n) of Mn(III) ions were obtained using the following equation:

(1) $$2.828\;\sqrt{\chi_{m}T}=\mu_{eff}=\sqrt{n\left(n+2\right)}$$

As shown in Figure 3h, the n of Mn(III) increased from 1.85 (*t*_2*g*_^4^ *e_g_* ^0^,*S* = 1)*in*δ − *MnO*_2_ *to*2.49(*t*_2*g*_^3^ *e_g_* ^1^,*S* = 2) in *ε*‐MnO_2_, suggesting its high‐spin state. For Mn‐based species, high‐spin Mn^3+^ normally has only one electron occupying one *e*_g_ orbital (*e*_g_1), which leads to the asymmetric occupation state of *the e*_g_ orbitals. In addition, the Mn nuclei are shielded in different directions by electrons distributed in *the d*_x2‐y2_ and *d*_z2_ orbitals.^[^ ^19^ ^]^ In this configuration, the overall *d* orbitals do not match the O_h_ symmetry of the octahedra, resulting in the elongation of the two longitudinal Mn–O bonds in octahedral MnO_6_, whereas the other four horizontal Mn–O bonds are shortened.^[^ ^16a^ ^]^ Such distortion in configuration matches well with the J–T effect, which causes the symmetry reduction of the MnO_6_ octahedron from O_h_ to D_4h_, accompanied by the elimination of degenerate orbitals, reduction of the system energy, and distortion of crystal structures.^[^ ^18b^ ^]^ Thus, it is very favorable for the dissociation and activation of oxygen atoms and provides a direct explanation for the excellent oxidase‐like activity of *ε*‐MnO_2_ nanozyme at low temperatures. It is worth mentioning that *γ*‐MnO_2_ has an effective magnetic moment (3.14 µeff) similar to that of *ε*‐MnO_2_ (3.34 *µ*_eff_), and the former also displayed comparatively cold‐adapted oxidase‐like activity. These results demonstrate that the spin state of MnO_2_ is indispensable for obtaining cold‐adapted enzymes.^[^ ^9^ ^]^

Based on this inference, density functional theory (DFT) calculations were performed to further predict the relationship between the spin states of MnO_2_ and cold‐adapted activity.^[^ ^22^ ^]^ It should be noted that the six MnO_2_ models, if in perfect crystal structure, are identical in spin state with comparable magnetic moments in the range of 2.9978–3.0001. (Table S2, Supporting Information) In fact, as suggested from the IR results of pyridine adsorption (Figure 3i), none of the synthesized MnO_2_ had a perfect crystalline style. Three typical signal peaks belonging to the *ν*1, *ν*12, and *ν*18 ring modes of vacancy‐style oxygen were identified at 1014, 1040, and 1069 cm^−1^ in the IR spectra of the MnO2 samples.^[^ ^23^ ^]^ These characteristic peaks were specifically detected for *ε*‐MnO_2_ with the strongest intensity, which is indicative of its maximum abundance of oxygen vacancies. The magnetic moment variation of *ε*‐MnO_2_ was then calculated by randomly adding different numbers of oxygen vacancies. As shown in Table S3 (Supporting Information), no matter where it is positioned, each additional oxygen vacancy in *ε*‐MnO_2_ increases the magnetic moment by +2, implying that the high‐spin state is induced by oxygen vacancies. Consequently, the six Mn‐O bonds of MnO_6_ of equal length (1.93 Å) were distorted when one oxygen atom, irrespective of position, was removed (Figure S10, Supporting Information), which is the elongation of the two longitudinal Mn‐O bonds (to 1.97 Å), along with the shrinkage of two horizontal Mn‐O bonds (to 1.91 Å) (Figure 3j). These are typical characteristics of J–T distortion caused by high‐spin Mn^3+^ in *ε*‐MnO_2_. In addition, according to a comparison between octahedral Mn^3+^O_6_ and Mn^4+^O_6_ (Figure S11, Supporting Information) with respect to the differential charge densities, we found that only Mn^3+^O_6_ has an occupied *d*_z_^2^ orbital,^[^ ^19^ ^]^ as reflected by the yellow regions beside the central Mn^3+^ cation, while high‐spin Mn^4+^ cannot, which demonstrates that only Mn^3+^ in the high‐spin state can induce J–T distortion.

Based on this understanding, the energy of the oxidation reaction at the *ε*‐MnO_2_ surface was calculated using TMB as the reducing agent at various temperatures.^[^ ^24^ ^]^ The activation of O_2_ on *ε*‐MnO_2_ is shown in Figure S12 (Supporting Information). In the first step, O_2_ is absorbed onto *ε*‐MnO_2_ to form MnO_2_^*^O_2_, which accepts a H^+^ ion and loses one electron to form MnO_2_^*^OOH. Then, MnO_2_^*^OOH receives an H^+^ ion to generate MnO_2_^*^O, followed by the formation of MnO_2_^*^OH due to H^+^ ion acceptance. Finally, MnO_2_^*^OH and H^+^ ions form MnO_2_ and H_2_O, respectively. The first step of oxygen adsorption determines the electron transfer activity from the active center to the adsorbed intermediate, where *ε*‐MnO_2_ catalyzes the oxidation of TMB to generate _ox_TMB. As shown in the energy profile diagram (Figure 3k), high‐spin *ε*‐MnO_2_ lowered the energy barrier for both O_2_ adsorption and the rate‐determining step (from MnO_2_^*^OOH to MnO_2_^*^O), which agrees well with the above‐mentioned results and provides thermodynamic evidence that the reaction is favorable for high‐spin *ε*‐MnO_2_. Additionally, the high‐spin *ε*‐MnO_2_ presented almost identical energy profiles for the reactions conducted at temperature of −20, 0, and 25 °C, which provided solid evidence for its stable activity over a wide temperature range. In contrast, low‐spin *ε*‐MnO_2_ was challenged with a much higher energy barrier to realize the reaction at a low temperature (Figure S13, Supporting Information). This could be explained by the fact that the J–T distortion induced by high‐spin Mn(III) affords *ε*‐MnO_2_ with both better dissociation toward key intermediates and lower activation energy barriers, thus empowering them with robust cold‐adapted nanozyme activity.

### 2.3 The Cryogenic Activity of MnO_2_ Nanozyme is Regulated by the Spin State

**Figure 4:**
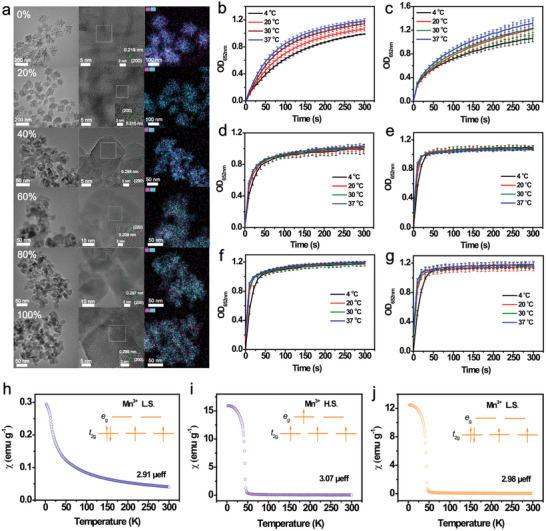
a) TEM and HRTEM images and EDS elemental maps of the Mn_3_O_4_ precursor treated at different oxygen partial pressures. Kinetic curves of TMB oxidation catalyzed by b) Mn_3_O_4_‐0%, c) Mn_3_O_4_‐20%, d) *δ*‐MnO_2_‐40%, e) *δ*‐MnO_2_‐60%, f) *δ*‐MnO_2_‐80%, and g) *δ*‐MnO_2_‐100% at different temperatures. Magnetic susceptibility plots of h) Mn_3_O_4_‐0%, i) *δ*‐MnO_2_‐40%, and j) *δ*‐MnO_2_‐80% (L.S. represents low spin, H.S. represents high spin).

To verify this hypothesis, the spin‐state reconfiguration of Mn^3+^ was further evaluated by utilizing Mn_3_O_4_ as a precursor, which was processed by sintering at 200 °C in a mixed atmosphere at various O_2_ partial pressures (0%, 20%, 40%, 60%, 80%, and 100% O_2_).^[^ ^25^ ^]^ The Mn_3_O_4_ samples exhibited a gradual lightened color (Figure S14a, Supporting Information) with a slight variation in morphology (**Figure** **4**a), along with an increase in the oxygen partial pressure. The XRD patterns (Figure S14b, Supporting Information), together with the HRTEM images (Figure 4a), revealed that the crystalline structure of Mn_3_O_4_ (JCPDS file: 24‐0735) was transformed into *δ*‐MnO_2_ with a Hausmannite tetragonal crystal structure (JCPDS file: 24‐0734) in all cases. Additionally, high‐valent manganese (Mn^3+^, Mn^4+^) was gradually converted to a low‐valent species (Mn^2+^) (Figure S14c,d, Supporting Information), which was coupled with a decrease in the AOS with a minimum AOS achieved at an oxygen partial pressure of 60% (Table S4, Supporting Information). In addition, the IR spectra of pyridine adsorption (Figure S15, Supporting Information) showed that *δ*‐MnO_2_‐40% has a maximum intensity of oxygen vacancies, which is beneficial for realizing a high‐spin state.^[^ ^26^ ^]^ This was confirmed by the calculated magnetic moment, showing a higher value of 3.07 *µ*_eff_ obtained for *δ*‐MnO_2_‐40% (Figure 4h–j). As desired, *δ*‐MnO_2_‐40% exhibited a maximum cold‐adapted activity compared with the other samples (Figure 4b–g), which again confirms that the high‐spin Mn^3+^ is the key factor determining the excellent cold‐adapted activity of MnO_2_.

### 2.4 CS Conversion at Low Temperature

Biomass, especially lignocellulosic biomass, is characterized by its carbon neutrality, abundance of chemical functionalities, renewability, and potential for sustainable production of chemicals, fuels, and materials.^[^ ^27^ ^]^ However, current strategies face great challenges in realizing efficient degradation under mild conditions.^[^ ^28^ ^]^ Given the superior cold‐adapted nanozyme activity of *ε*‐MnO_2_, together with the catalytic oxidation ability of MnO_x_ toward numerous substrates,^[^ ^11^, ^29^ ^]^ it is expected to be a promising alternative to solve the above‐mentioned issues.

**Figure 5:**
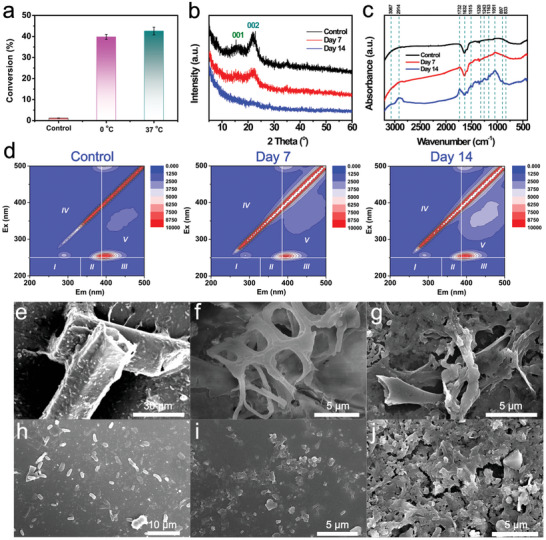
a) Conversion percentages of CS treated with *ε*‐MnO_2_ at different temperatures. b) XRD patterns, c) FTIR spectra, and d) 3D fluorescence contour maps of CS treated with *ε*‐MnO_2_ overtime at 0 °C. SEM images of CS treated with *ε*‐MnO_2_ for e) 0, f) 7, and g) 14 days at 0 °C. High‐resolution SEM images of CS treated with *ε*‐MnO_2_ for h) 0, i) 7, and j) 14 days at 0 °C.

Therefore, the catalytic performance of *ε*‐MnO_2_ in lignocellulose degradation was evaluated using corn straw (CS) as a model.^[^ ^30^ ^]^ After coincubation with *ε*‐MnO_2_ nanozyme for 14 days at different temperatures, lignin was degraded with a conversion rate of ≈40% at both 0 and 37 °C (**Figure** **5**a), indicating the robust performance of *ε*‐MnO_2_ in lignocellulose transformation, especially under cold conditions. The structural changes of CS, including crystallinity, morphology, and chemical linkages, after treatment with *ε*‐MnO_2_ were systematically examined. As shown in the XRD data (Figure 5b), the characteristic peaks (at 16° and 22°) corresponding to the lattice planes of cellulose I in CS showed a downward trend in intensity as the incubation time increased and disappeared after 14 days of treatment.^[^ ^31^ ^]^ This is coupled with the emergence of numerous acid‐, phenol‐, and ketone‐containing compounds, which are clearly reflected in the FTIR pattern (Figure 5c). Similar indications were also observed in 3D excitation emission matrix fluorescence spectroscopy (3DEEM),^[^ ^32^ ^]^ which showed the emergence of high‐purity humic acid‐like substances in the products (Figure 5d). Additionally, the fluorescence peaks in the region of Em = 275–310 nm and Ex = 245–265 nm representing phenols, indoles, mono‐and polyaromatic hydrocarbons, and lignin degradation products (lignin phenols, vanillic acid, syringic acid, etc.) were greatly enhanced in intensity after treatment. It can be concluded that CS decomposes and transforms into small molecules. The SEM characterization clearly depicted the morphological change of CS from an intact rod into a porous skeleton with the appearance of holes on the surfaces during the treatment process (Figure 5e–g). In addition, the large particles cracked into small pieces (Figure 5h–j). These findings, in combination, firmly demonstrated that *ε*‐MnO_2_ could efficiently degrade CS under both high and low temperatures, providing a novel strategy to utilize lignocellulose under mild conditions.

## 3 Conclusion

In summary, we developed a novel cold‐adapted nanozyme of *ε*‐MnO_2_ that displays extremely stable oxidase‐like activity in the temperature range of −20 to 45 °C. Experimental evaluations along with theoretical calculations revealed that the key factor determining the cold‐adapted property is the spin states of Mn in *ε*‐MnO_2_, which induces the J–T effect to benefit both oxygen dissociation and activation. Additionally, the cold‐adapted property of *δ*‐MnO_2_ with relatively poor low‐temperature activity was successfully optimized through changing the spin states of Mn. This again proves the decisive role of the spin state on the cold‐adapted performance. Finally, a mild strategy for corn straw degradation at low temperatures was developed based on the superior cold‐adapted activity of *ε*‐MnO_2_, showing significant structural fragmentation and corresponding transformation into small molecules. This study provides guidance for the rational design of cold‐adapted nanozymes based on current inorganic materials without flexible configurations. The strategy developed for corn straw degradation not only broadens the application of nanozymes in wide fields, but also provides new insights into biomass utilization under mild conditions.

## Conflict of Interest

The authors declare no conflict of interest.

## Supporting information



Supporting Information

## Data Availability

The data that support the findings of this study are available in the supplementary material of this article.
