# Supplementary material for: High‐Spin States of Manganese(III) Enable Robust Cold‐Adapted Activity of MnO2 Nanozymes
Source: Adv Sci (Weinh). 2024 Dec 16;12(6):2415477. doi: 10.1002/advs.202415477 (PMC11809346; doi:10.1002/advs.202415477)
Supplement: Supplementary file 1 — Supporting Information [file ADVS-12-2415477-s001.docx]

**High -spin States of Manganese(**III**) Enable Robust Cold-adapted Activity of MnO_2_ Nanozymes**

*Qing Tian ^a^, Yao Chen ^a,^ *, Shuaiqi Huangfu ^a^, Ge Kang ^a^, Haoyu Wang ^a^, Huile Liu ^a^, Xuejing Wang ^a^, Aipeng Li ^b^, Kelong Fan ^c,^ *, and Lianbing Zhang ^a,^ **

1. Q. Tian, Y. Chen, S.Q. Huangfu, G. Kang, H.Y. Wang, H.L. Liu, X.J. Wang, and Prof. L.B. Zhang

School of Life Sciences, Northwestern Polytechnical University, 127 Youyi Road, Xi’an, 710072, China

[b] A. Li

Xi’an Key Laboratory of C1 Compound Bioconversion Technology, School of Chemical Engineering and Technology, Xi’an Jiaotong University, Xi’an 710049, China

[c] Prof. K.L. Fan

CAS Engineering Laboratory for Nanozyme, Institute of Biophysics Chinese Academy of Sciences, 15 Datun Road, Beijing, 100101, China

Email: fankelong@ibp.ac.cn; chenyao@nwpu.edu.cn and lbzhang@nwpu.edu.cn

**Experimental details**

**Chemicals and Materials.** Manganese(II) nitrate tetrahydrate (Mn(NO_3_)_2_∙4H_2_O), manganese (II) sulfate monohydrate (MnSO_4_∙H_2_O), H_2_O_2_ (30%), acetic acid, sodium acetate, nitric acid (HNO_3_), sulfuric acid (H_2_SO_4_), ammonium persulfate ((NH_4_)_2_S_2_O_8_), potassium permanganate (KMnO_4_), N,N-dimethylformamide (DMF), dimethyl sulfoxide (DMSO), polyvinyl pyrrolidone (PVP), ethanol, and methanol were all obtained from China National Medicines Corporation Ltd (China). Oleic acid, oleylamine, tert-butanol (DOB), 3,3',5,5'-tetramethylbenzidine (TMB), cerium(III) nitrate hexahydrate (Ce(NO_3_)_3_∙H_2_O), *β*-carotenoids, 1,4-benzoquinone, and lithium acetate (LiAc) were purchased from Shanghai Aladdin Biochemical Technology Co., Ltd. Tannic acid, sodium hydrogen carbonate (NaHCO_3_), potassium chloride (KCl), and citric acid were purchased from Med Chem Express (MCE, USA). Horseradish peroxidase (HRP, ≥ 300 U/mg) was obtained from Sangon Biotech Co., Ltd. (China). Milli-Q ultrapure water (>18 MΩ∙cm) was used in all experiments. All chemicals were reagent grade and used as received without further purification.

**Synthesis of MnO nanosphere (MnO NSs).** According to previous reports, MnO NSs with an average diameter of approximately 10 nm were successfully synthesized. Typically, manganese acetate and tannic acid (M_manganese acetate_/M_tannic acid_ = 1:2.8) were introduced into Milli-Q ultrapure water at room temperature. The mixture was then transferred to a 100-mL Teflon-lined autoclave container and heated for 2 h at 120°C. Subsequently, the mixed solution was centrifuged at 4000 rpm for 10–15 min to remove large flocculent materials and further purified by dialysis against distilled water. The purified MnO NSs were lyophilized for further use.

**Synthesis of Mn_2_O_3_ octahedra.** Mn_2_O_3_ samples were obtained by referring to previous reports. Typically, 4 mmol Mn(NO_3_)_2_∙4H_2_O was dissolved into 13 mL absolute alcohol at room temperature. The mixture was then moved into a 23-mL autoclave and heated for 12 h at 100°C. Subsequently, the reaction solution was allowed to cool naturally to room temperature and centrifuged at 10,000 rpm for 10 min to collect sediment. Finally, the resulting product was washed three times with ddH_2_O and ethanol to remove residual Mn ions.

**Synthesis of Mn_3_O_4_ nanoflowers (Mn_3_O_4_ NFs).** In a typical procedure, 6.3 mmol KMnO_4_ was dissolved in 500 mL ddH_2_O following by rapid stirred for 30 min at room temperature. Subsequently, 10 mL of oleic acid was added into the reaction solution, and a steady emulsion was formed. After the mixture aged for 5 h, brown-black products were collected and washed several times with ddH_2_O and alcohol to remove residual reactants. The sediment was dried in air at 50°C for 12 h to obtain the precursor, which was calcined in air at 200°C for 5 h to give the Mn_3_O_4_ NFs.

**Synthesis of Mn_3_O_4_ NFs with different concentrations of oxygen vacancies.** Mn_3_O_4_ samples with different oxygen vacancy concentrations were synthesized by sintering under various oxygen partial pressures. The MnO_2_ NFs were sintered at 0%, 20%, 40%, 60%, 80%, and 100% (oxygen partial pressure), and the resulting products were respectively labeled as Mn_3_O_4_ NFs-0%, Mn_3_O_4_ NFs-20%, Mn_3_O_4_ NFs-40%, Mn_3_O_4_ NFs-60%, Mn_3_O_4_ NFs-80%, and Mn_3_O_4_ NFs-100%.

**Synthesis of MnO_2_ samples.** *α-*, *β-*, *δ-*, and *γ*-MnO_2_ were fabricated by a hydrothermal method according to previous reports. For *α*-MnO_2_, the detailed procedure was as follows. MnSO_4_ (3 mmol) and KMnO_4_ (8 mmol) were mixed in 80 mL distilled water. After adding 2 mL of 68% HNO_3_, the solution was stirred magnetically for 30 min to obtain a homogeneous solution. The solution was then transferred into a Teflon stainless-steel autoclave and continuously heated at 100°C for 24 h to form a black product. The resulting product was collected by centrifugation and dried at 80°C for 12 h to obtain *α*-MnO_2_.

The preparation processes of *β*-MnO_2_, *δ*-MnO_2_, and *γ*-MnO_2_ were similar to that of *α*-MnO_2_ with the following differences. For *β*-MnO_2_, 10 mmol MnSO_4_ and 10 mmol (NH_4_)_2_S_2_O_8_ were mixed, and the autoclave was heated at 140°C for 12 h. For *γ*-MnO_2_, 20 mmol MnSO_4_ and 20 mmol (NH_4_)_2_S_2_O_8_ were mixed, and the autoclave was heated at 90°C for 24 h. For *δ*-MnO_2_, 1.6 mmol MnSO_4_ and 10 mmol KMnO_4_ were mixed, and the autoclave was heated at 160°C for 24 h.

For *ε*-MnO_2_, 2 mmol MnSO_4_ and 20 mmol NaHCO_3_ were added into 70 mL distilled water. After complete dissolution, 7.0 mL of ethyl alcohol was added into the MnSO_4_ solution, and NaHCO_3_ solution was added dropwise into the MnSO_4_ solution. After stirring for 3 h at room temperature, the white products were collected by centrifugation, dried at 80°C for 12 h, and calcined at 400°C for 6 h to obtain *ε*-MnO_2_.

For *λ*-MnO_2_, 15 mmol LiAc and 28 mmol Mn(Ac)_2_.4H_2_O were dissolved in 70 mL distilled water, and 4 mL of 68% HNO_3_ was added to adjust the pH value to 1–3. Subsequently, 33 mmol citric acid was added into the mixed solution and stirred magnetically to form a homogeneous solution. The solution was transferred to a Teflon stainless-steel autoclave and heated for 24 h at 160°C. The obtained sol was stirred at 80°C to obtain a gel, which was dried at 120°C for 12 h and calcined at 750°C for 6 h. The resulting black powder was immersed in H_2_SO_4_ solution for 12 h to remove residual ions and organic matter. Finally, *λ*-MnO_2_ was obtained after centrifugation and drying.

**Synthesis of** ***ε*-MnO_2_ with different morphologies**

**Synthesis of massive *ε*-MnO_2_**. MnSO_4_•H_2_O (1 mmol) and (NH_4_)_2_SO_4_ (1 mmol) were dissolved into a mixture of ddH_2_O (70 mL) and ethyl alcohol (7 mL). Meanwhile, 10 mmol NaHCO_3_ was dissolved into 20 mL ddH_2_O. NaHCO_3_ solution was then added dropwise into the mixture of ddH_2_O and ethyl alcohol. After stirring for 7 h at 50°C, the white products were collected by centrifugation and washed several times with ddH_2_O. Finally, the products were dried at 80°C for 12 h and then calcined at 400°C for 6 h to obtain massive *ε*-MnO_2_.

**Synthesis of spherical *ε*-MnO_2_**. MnSO_4_•H_2_O (1 mmol) was dissolved into a mixture of ddH_2_O (70 mL) and ethyl alcohol (7 mL). Meanwhile, 10 mmol of NaHCO_3_ was dissolved into 20 mL of ddH_2_O. After mixing the two solutions, the reaction solution was stirred for 10 min and allowed to rest for 3 h at room temperature. The obtained white precipitate was collected and calcined for 6 h at 400°C.

**Synthesis of oval-shaped** ***ε*-MnO_2_**. KMnO_4_ (3 mmol), D-glucose monohydrate (3 mmol), and tartaric acid (1 mmol) were dissolved in ddH_2_O (70 mL). After stirring for 10 min, the mixture was heated for 10 h at 150°C. The formed products were collected by centrifugation, washed several times with ddH_2_O, and calcined for 6 h at 400°C to obtain oval-shaped *ε*-MnO_2_.

**Synthesis of lamellar *ε*-MnO_2_.** Mn(CH_3_COO)_2_•4H_2_O (1 mmol) and 0.80 g polyvinyl pyrrolidone (PVP) were dissolved into methanol (80 mL). After transferring the solution into a Teflon stainless-steel autoclave and heated for 12 h at 120°C, the solution was cooled to room temperature, and the products were collected by centrifugation and washed several times with ddH_2_O and alcohol. The obtained products were calcined for 10 h at 650°C, and the black powder was then immersed in H_2_SO_4_ solution for 12 h to remove residual ions and organic matter. Finally, the *ε*-MnO_2_ nanosheets were obtained after centrifugation and drying.

**Synthesis of flower-shaped *ε*-MnO_2_.** KMnO_4_ and MnSO_4_ (molar ratio = 2:1) were dissolved in ddH_2_O, and 10 mL of 68% HNO_3_ was added to adjust the pH value to 1–3. The mixed solution was stirred magnetically for 24 h at room temperature, and the dark brown products were obtained by centrifugation, washing, and drying.

**Synthesis of Pt nanoparticles (Pt NPs).** Pt NPs were prepared using a typical solvothermal method according to a previous report. Briefly, H_2_PtCl_6_•H_2_O (6.55×10^−5^ mol) and PVP (1.31×10^−3^ mol) were dissolved in a mixture of ethanol and ddH_2_O (v/v = 1:1). The reaction solution was heated for 3 h at 150°C under vigorous stirring. The formed black precipitate was collected and dried at 50°C overnight.

**Sythesis of CeBTC.** Ce(NO_3_)_3_•6H_2_O (0.5 mmol) and BTC (0.5 mmol) were mixed in an ethanol/water solution (50 mL, v/v = 1:1) under vigorous stirring at room temperature. The reaction mixture was then heated for 2 h at 90°C, and the white product was collected by centrifugation. Finally, the precipitate was washed several times with ddH_2_O and ethanol and further dried at 60°C overnight.

**Characterizations.** The morphologies and microstructures of the samples were characterized by double C-s-corrector transmission electron microscopy (Tecnai G2 F30 S-TWIN, FEI, USA). X-ray diffraction (XRD) patterns were collected using a D8 DISCOVER A25 diffractometer over the 2*θ* range from 5° to 80° with Cu Kα radiation. The surface electronic states of samples were evaluated by X-ray photoelectron spectroscopy (XPS) using a Thermo ESCALAB 250 spectrometer with a monochromatic Al K*α* X-ray source. Electron spin resonance (ESR) measurements were carried out using a Bruker ESR spectrometer (EMXPLUS10/12, Germany). Fourier-transform infrared (FTIR) spectroscopy was performed using a Nicolet iS50 Spectrometer (Thermo Fisher, USA). The FTIR spectra were recorded in the region of 4000–600 cm^−1^ at a resolution of 2 cm^−1^. The ultraviolet–visible (UV-vis) absorption spectra and the time-dependent absorbance spectra were recorded using a microvolume UV-Vis spectrophotometer (Nanodrop one, Thermo Scientific, USA). Electrochemical experiments were conducted on a CHI760E electrochemical workstation (CH Instrument Co., USA). Zero-field-cooled (ZFC) and field-cooled temperature-dependent magnetic susceptibility values were measured using a physical property measurement system (PPMS, Quantum Design PPMS-9T) to reveal the electron spin configurations of the MnO_2_ samples. Oxygen temperature programming desorption (O_2_-TPD, Micromeritics AutoChem II 2920, USA) and H_2_ temperature programmed reduction (H_2_-TPR, Micromeritics AutoChem II 2920, USA) experiments were performed to analyze the oxygen storage capacity. Three-dimensional fluorescence contours were measured using a Leng Guang Tech F97XP spectrofluorometer.

**Oxidase-mimicking activity assay.** The oxidase-mimicking activities of the MnO_x_ samples were measured using the chromogenic reaction of TMB. This reaction produces a blue signal with two specific absorption peaks located at wavelengths of 370 and 652 nm. Typically, 5 μL catalyst (2 mg/mL) and 10 μL TMB (25 mM) were sequentially added into HAc-NaAc buffer (0.2 M, pH 3.6), and the final volume was fixed to 1 mL. The absorbance of the reaction solution was then measured at 652 nm using a UV-Vis spectrophotometer.

**Cold-adapted enzyme mimicking activity.** The performance of MnO_x_ as a cold-adapted enzyme was evaluated by referring to our previous work. In detail, the oxidase-like activities of MnO_x_ were measured using a UV-Vis spectrophotometer at different temperatures (4°C, 20°C, 30°C, and 37°C) based on a kinetic model. The oxidase-like activities of HRP, Pt NPs, and CeBTC at different temperatures were also measured for comparison. The absorbances at 652 nm of the reaction system catalyzed by *ε*-MnO_2_ at a series of temperatures (−20°C, −10°C, 0°C, 20°C, and 45°C) were also detected after incubation for 5 min.

**Detection of oxygen storage capacity.** O_2_-TPD experiments were conducted to analysis the oxygen storage capacity. The sample (75 mg) was placed in a U-shaped quartz tube, and the temperature was increased from room temperature to 150°C at 10°C/min for pretreatment. The tube was then purged with flowing helium (50 mL/min) for 1 h and cooled to 50°C. Subsequently, the tube was purged with a mixture of 10% O_2_/He (50 mL/min) for 1 h until reaching saturation. The physically adsorbed O_2_ on the surface of the sample was then removed by purging for 1 h with flowing He (50 mL/min). Finally, the desorbed gas was detected using a TCD detector under a heating rate of 10°C min and flowing He (50 mL/min).

H_2_-TPR was carried out to measure changes in the H_2_ content in the gas stream. The sample (75 mg) was placed in a U-shaped quartz tube and heated from room temperature to 150°C at a rate of 10°C/min for pretreatment. The tube was then purged by flowing He (50 mL/min) and cooled to 50°C. A 10% H_2_/Ar mixture (50 mL/min) was injected for 1 h until achieving baseline stabilization. Finally, the sample was heated to 700°C at a rate of 10°C/min under 10% H_2_/Ar mixture, and the desorbed reduction gas was detected by TCD.

**Magnetic characteristic analysis.** The magnetic properties of the catalysts were studied using a physical property measurement system (PPMS, Quantum Design PPMS-9T). For ZFC and field-cooled measurements, vacuum-dried samples were demagnetized at 293 K by setting an initial field of 300 kOe and decreasing the field stepwise to zero by oscillating at 200 Oe/s. Samples were cooled to 2 K using a cryocooler-based cooling system at zero field. An external field of 300 Oe was then applied, and the samples were heated to 300 K at 1 K/min and again cooled to 2 K at 5 K/min in the 300-Oe field. The magnetization M was measured by vibrating the samples at 40 Hz. One data point was delivered for M measured within 1 s (averaging time). Hysteresis measurements were carried out at 20 and 300 K for each sample by cycling the applied field from −40,000 to 40,000 Oe at a rate of 5 Oe/s.

**Computational methods.**

The Vienna Ab initio Simulation Package software was used for all spin polarized DFT calculations. The projector increased wave (PAW) potentials were employed for electron-ion interaction, and the generalized gradient approximation (GGA) method with the Perdew, Burke, and Ernzerh (PBE) of functional was used for exchange and correlation potential. The DFT-D3 method proposed by Grimme was incorporated to account for intermolecular van der Waals (vdW) interactions. An energy cut-off of 500 eV ensured accuracy. The effective U value of 3.5 eV for Fe was selected based on previous studies. The convergence criterion of total energy was set to 10^−5^ eV, and that of force on each atom was set to 0.02 eV Å^−1^. The Monkhorst-Pack grid of 3× 3 × 1 was sampled in the Brillouin zone. The slab was modeled with a vacuum layer of 15 Å to separate the slabs along the perpendicular Z-direction. The Gibbs free energy change (ΔG) for each elemental step of O_2_ reduction was calculated via following formula: ΔG = ΔE + ΔE_ZPE_ – TΔS, where ΔE, ΔE_ZPE_ and ΔS are the electronic energy, zero-point energy and entropy difference between the reaction intermediates respectively.

**Electrochemical Characterization.** Cyclic voltammetry (CV) was performed using a CHI760E electrochemical workstation with a three-electrode electrochemical cell comprising a Pt wire counter electrode, a saturated Ag/AgCl reference electrode, and a glassy carbon electrode as the working electrode at room temperature. To prepare the working electrode, 6 mg of catalyst was dispersed in a 1 mL mixture of ethanol and 5% Nafion (v/v = 200:1) under sonication for 30 min to form a homogeneous catalyst ink. Subsequently, 10 μL of the mixture was placed into a glassy carbon rotating disk electrode with an area of 0.196 cm^−2^ and dried at room temperature. CV was performed in KCl-HAc solution (1.0 M, pH 3.6) as the supporting electrolyte. Finally, the working electrode was scanned cathodically at a potential scan rate of 0.02 V/s under air atmosphere. The onset potential and peak potential were identified using CHI760E electrochemical software.

**Catalytic oxidation of corn stalk by *ε*-MnO_2_**. Corn stalk powder (10.0 mg) was dispersed in 5 mL ddH_2_O followed by the addition of 1.0 mg *ε*-MnO_2_ nanozyme. The mixed solution was subjected to ultrasound for 5 min, and the solution was incubated at different temperatures (0°C and 37°C) under stirring. After incubation for 14 d, the powder was removed and collected by centrifugation. The conversion of corn stalk was calculated as follows:

Conversion = (1 − M_t_ / M) × 100%,

where *M*_t_ represents the mass of residual corn stalk, and *M* is the initial mass of the corn stalk.

**Statistical analysis.** Origin Pro Portable 8.5 (Origin Software) was used for statistical analyses. Unpaired Student’s two-sided t-test was employed to determine the differences between the two groups: ^*^*P* < 0.05, ^**^*P* < 0.01.


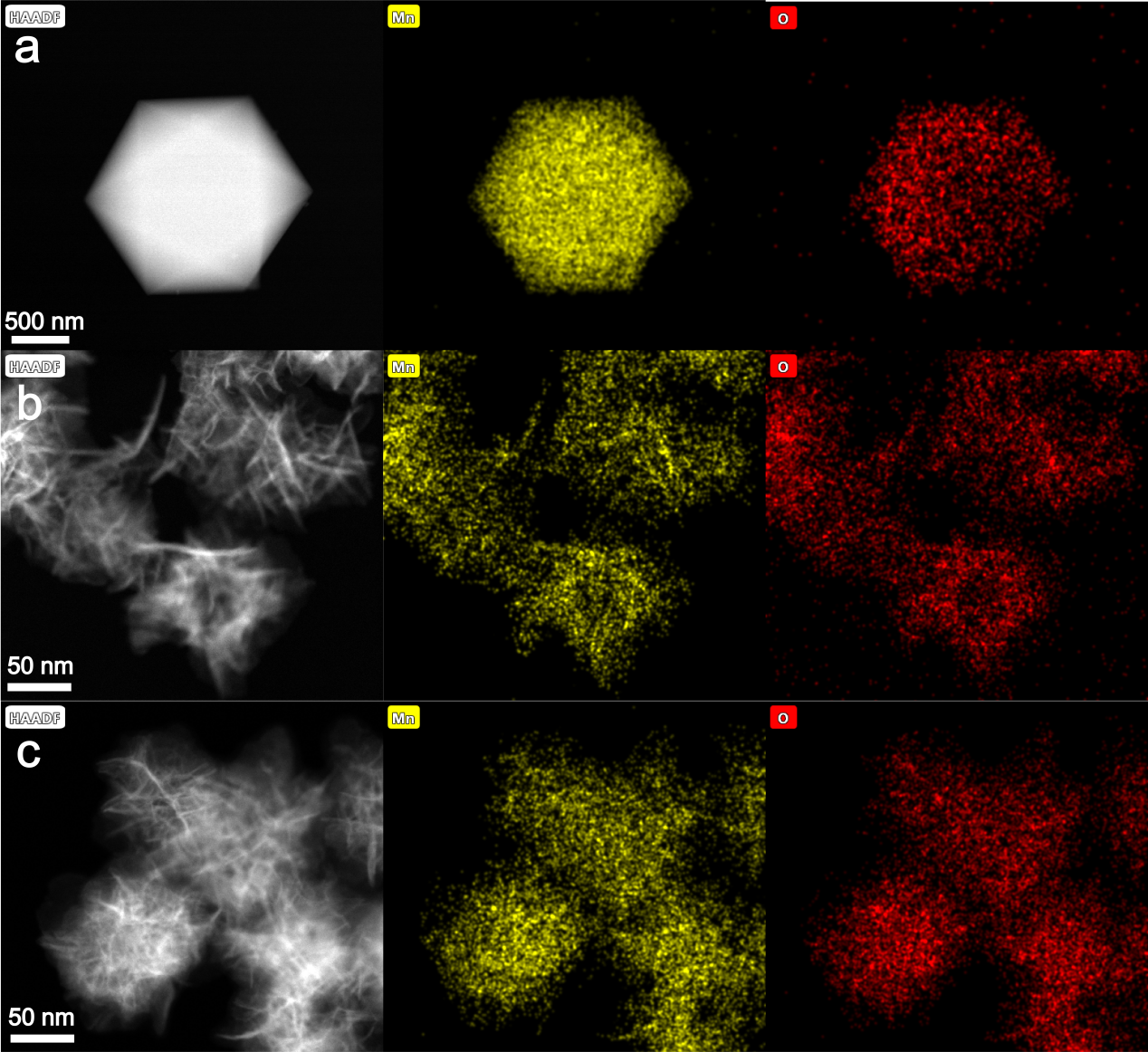


Fig. S1 Elemental mapping analysis of (a) Mn_2_O_3_, (b) Mn_3_O_4_ and (c) MnO_2_.


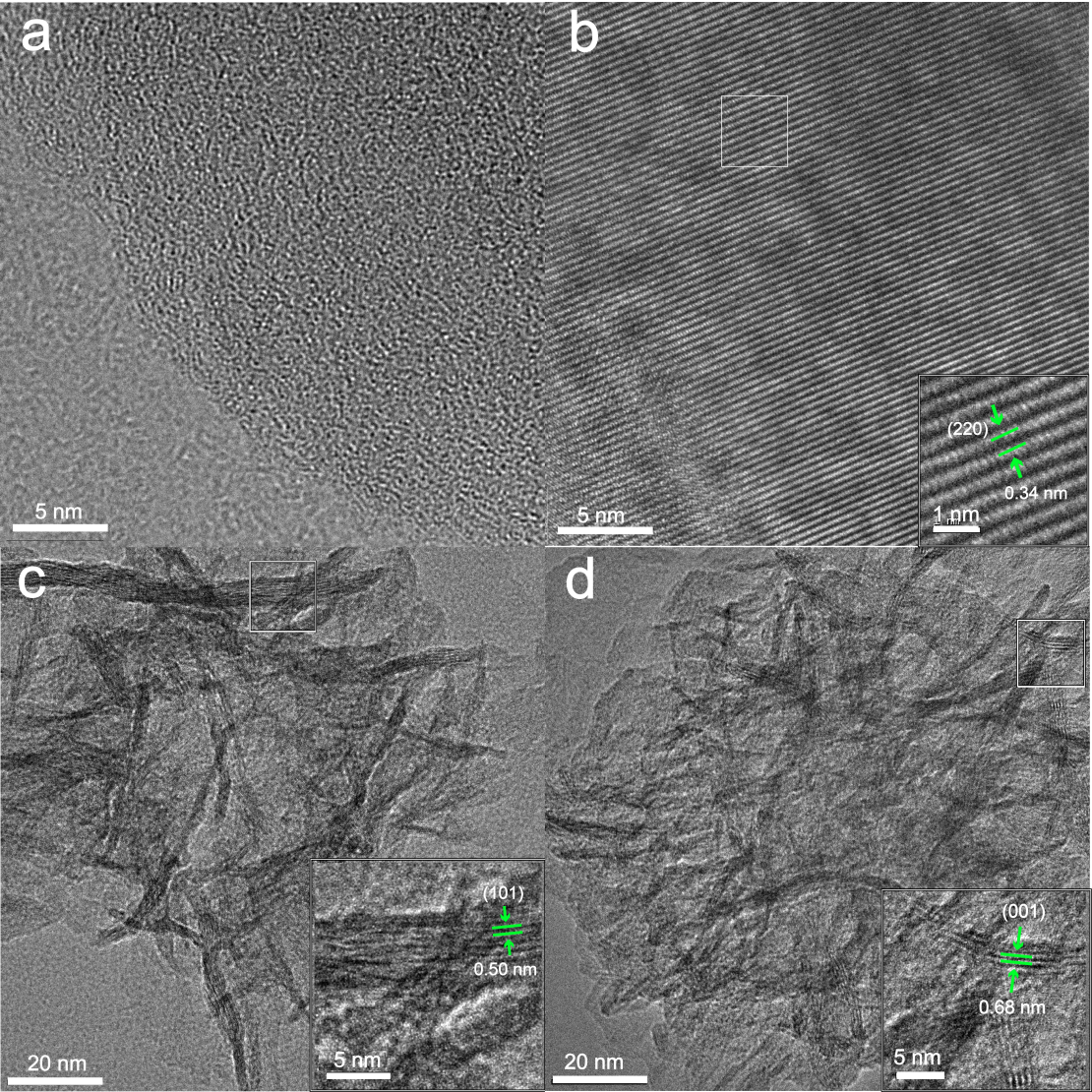


Fig. S2 HRTEM of (a) MnO, (b) Mn_2_O_3_, (c) Mn_3_O_4_ and (d) MnO_2_.


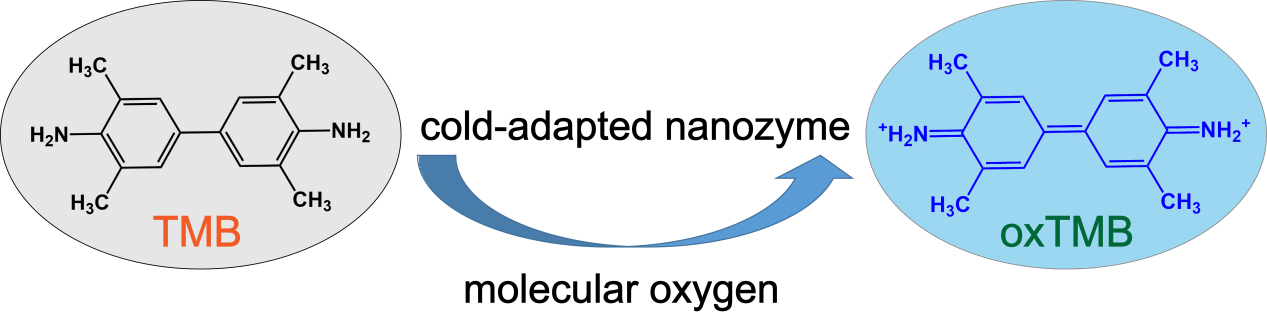


Fig. S3 Reaction mechanism of TMB catalyzed by oxidase mimics.


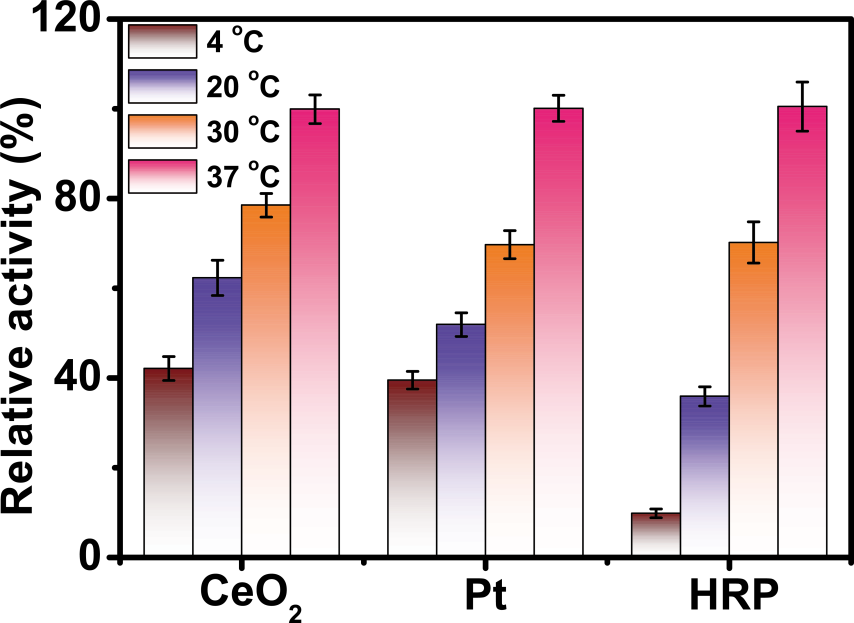


Fig. S4 Comparison of the relative activities of CeO_2_, Pt nanoparticles, and horse radish peroxidase (HRP) at various temperatures.


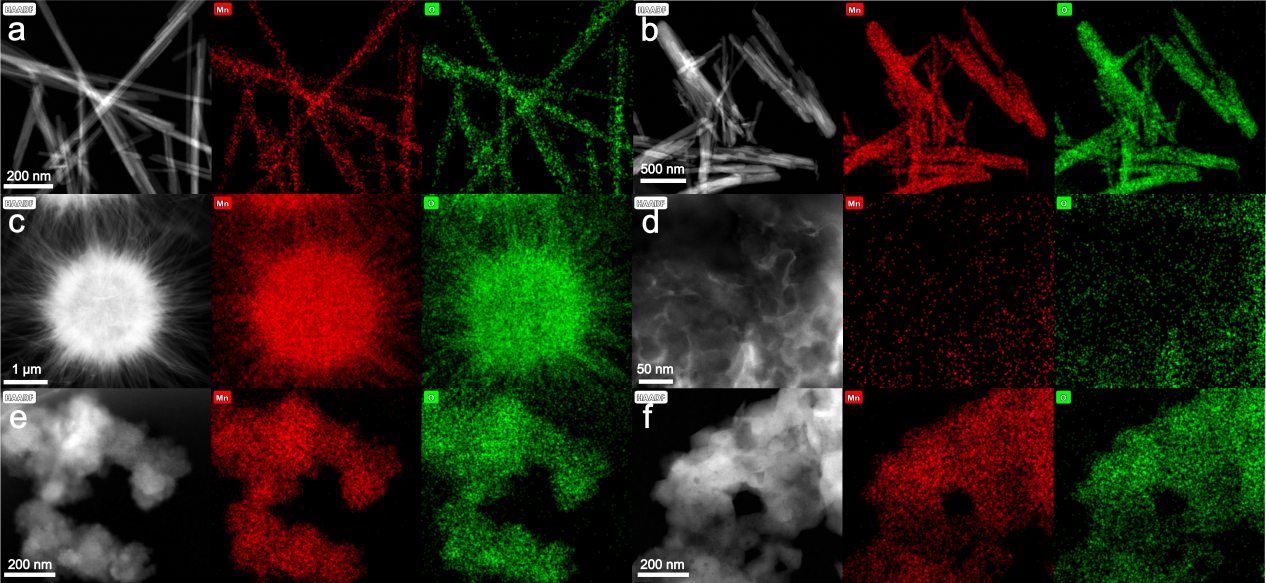


Fig. S5 Elemental mapping analysis of MnO_2_ with different crystal phases: (a) *α*-MnO_2_, (b) *β*-MnO_2_, (c) *γ*-MnO_2_, (d) *δ*-MnO_2_, (e) *ε*-MnO_2_, and (f) *λ*-MnO_2_.

Fig. S6 XRD patterns of MnO_2_ with different crystal phases.

Fig. S7 Kinetic curves of TMB oxidation catalyzed by *ε*-MnO_2_ and nMnBTC at different temperatures.


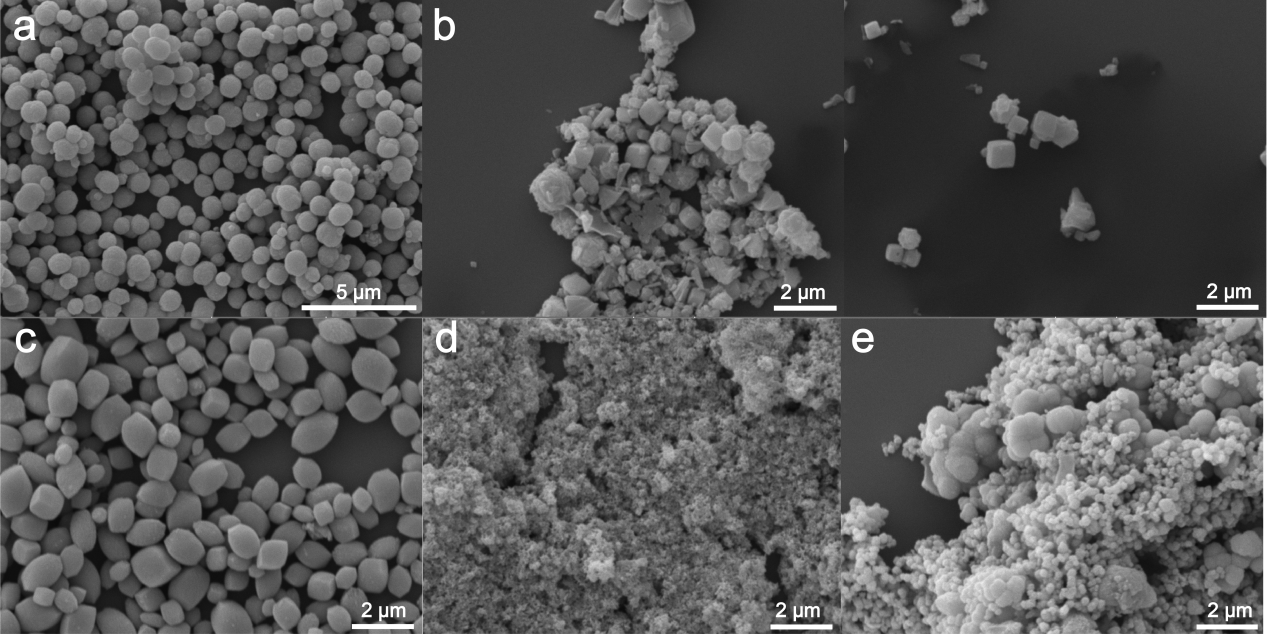


Fig. S8 SEM images of *ε*-MnO_2_ with different morphologies: (a) massive *ε*-MnO_2_, (b) oval-shaped *ε*-MnO_2_, (c) spherical *ε*-MnO_2_, (d) lamellar *ε*-MnO_2_, and (e) flower-shaped *ε*-MnO_2_.

Fig. S9 XRD patterns of *ε*-MnO_2_ with different morphologies are in good agreement with that previously reported for *ε*-MnO_2_ (JCPDS file: 30-0820)


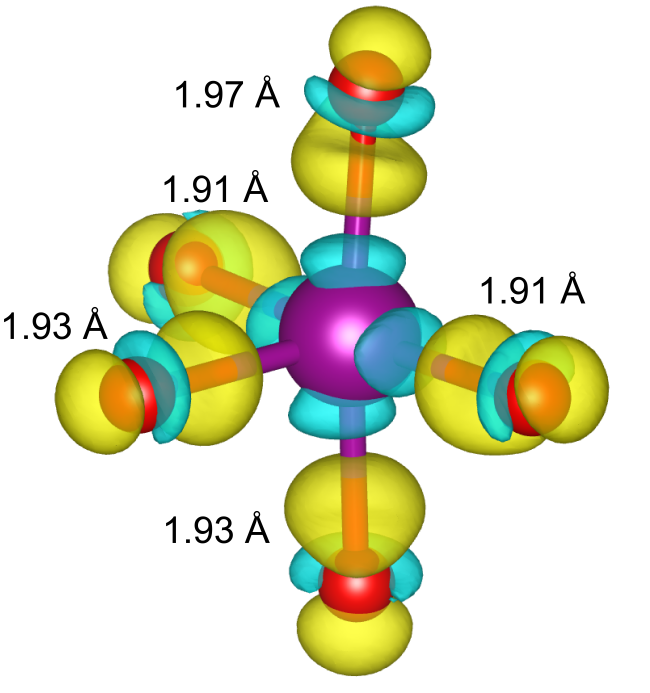


Fig. S10 The calculated differential charge densities of the octahedral Mn^3+^O_5_ with horizontal oxygen vacancy

.


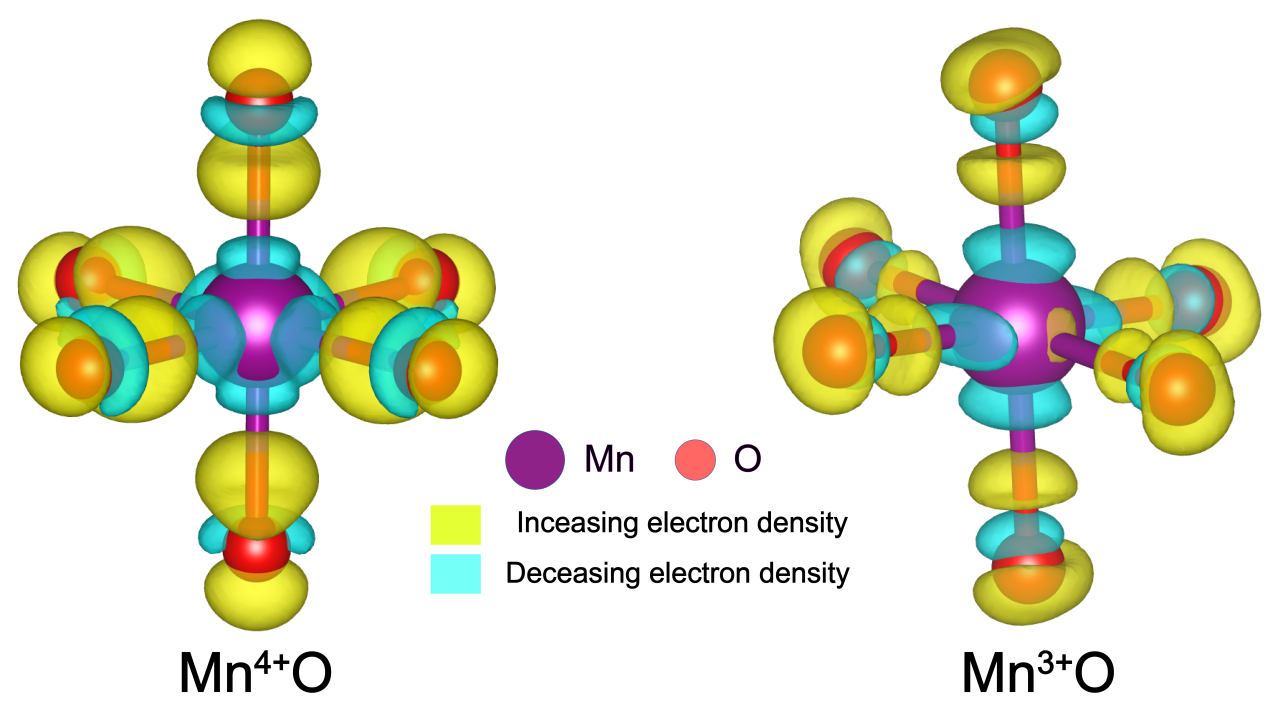


Fig. S11 The calculated differential charge densities of the octahedral Mn^4+^O_6_ and Mn^3+^O_6_.


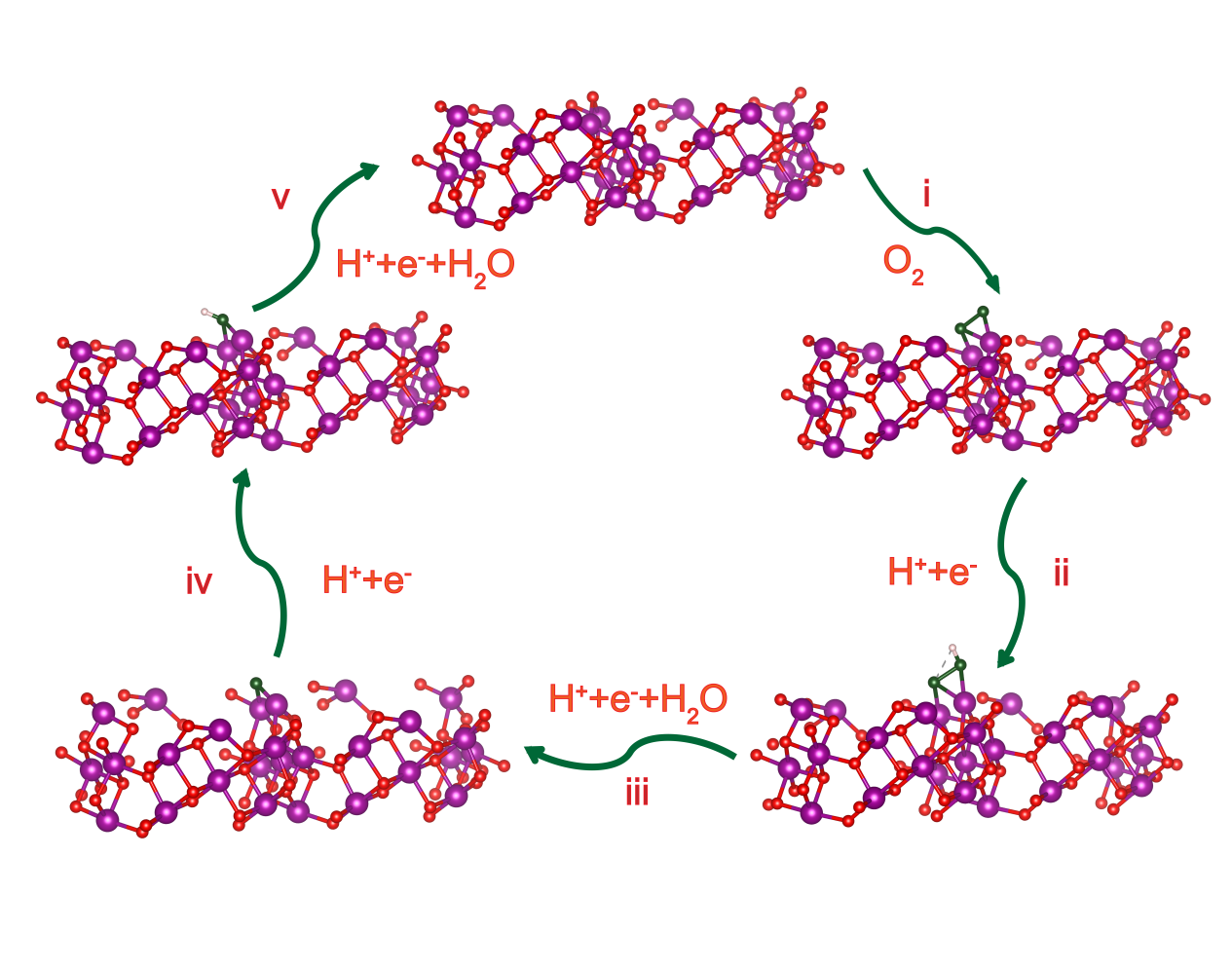


Fig. S12 Proposed reaction pathways of O_2_ reduction to H_2_O with optimized adsorption configurations on *ε*-MnO_2_ nanozyme. Purple, red, white balls represent Mn, O, and H, respectively. Green balls represent the adsorbed O atoms.


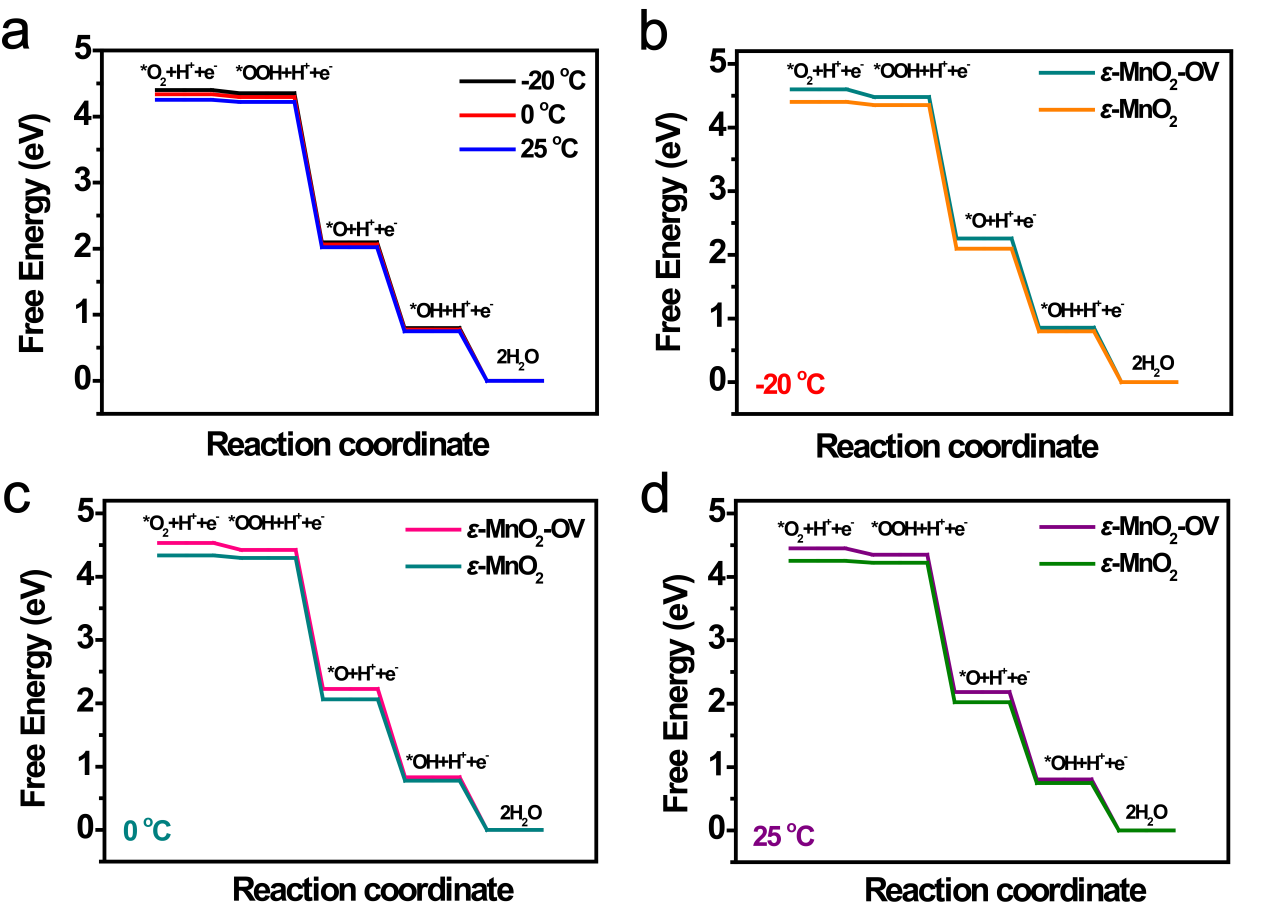


Fig. S13 The energy profile diagram for oxygen reduction reaction on *ε*-MnO_2_-OV and *ε*-MnO_2_ with TMB as reductant at different temperatures.


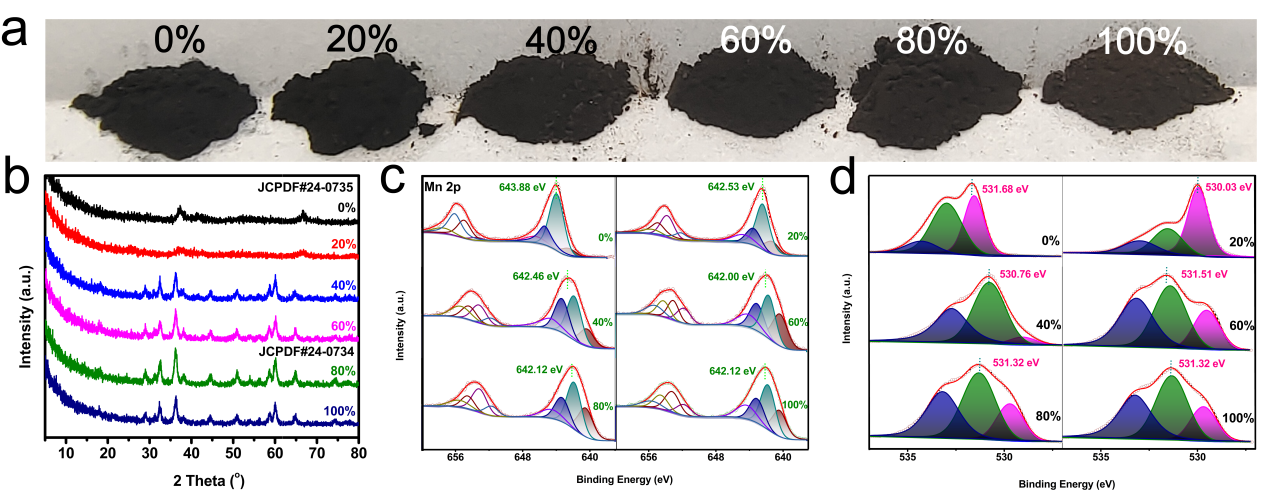


Fig. S14 (a) Appearances, (b) XRD patterns, and (c) Mn 2p and (d) O 1s XPS spectra of Mn_3_O_4_ precursors treated under different oxygen partial pressures at 200°C.

Fig. S15 Pyridine FTIR spectrum of the Mn_3_O_4_ treated with varied oxygen partial pressure.

| Sample | Mn 2p_3/2_ | | | O1s | | | AOS |
| --- | --- | --- | --- | --- | --- | --- | --- |
|  | Mn^2+^ (%) | Mn^3+^ (%) | Mn^4+^ (%) | O_latt_ (%) | O_sur_ (%) | O_ads_ (%) |  |
| *α*-MnO_2_ | 14.7 | 61.5 | 23.8 | 67.6 | 27.1 | 5.3 | 3.57 eV |
| *β*-MnO_2_ | 3.7 | 71.9 | 24.4 | 58.5 | 36.6 | 4.9 | 3.97 eV |
| *γ*-MnO_2_ | 12.9 | 74.2 | 12.9 | 63.1 | 31.5 | 5.4 | 3.49 eV |
| *δ*-MnO_2_ | 11.6 | 59.6 | 28.8 | 68.4 | 27.1 | 4.5 | 3.81 eV |
| *ε*-MnO_2_ | 29.4 | 34.7 | 35.9 | 44.3 | 45.9 | 9.8 | 3.33 eV |
| *λ*-MnO_2_ | 6.4 | 69.2 | 24.4 | 65.0 | 28.3 | 6.7 | 3.81 eV |

Table S1 Mn 2p XPS spectra analysis, O 1s XPS spectra analysis, and average oxidation state (AOS) of the synthesized MnO_2_ samples.

Table S2 The calculated spin state of MnO_2_ with different crystal structures

| samples | *α*-MnO_2_ | *β*-MnO_2_ | *β*-MnO_2_ | *δ*-MnO_2_ | *δ*-MnO_2_ | *λ*-MnO_2_ |
| --- | --- | --- | --- | --- | --- | --- |
| Spin state | 2.9983 | 2.9979 | 2.9994 | 2.9992 | 2.9982 | 3.0001 |

Table S3 The calculated magnetic moment and spin of *ε*-MnO_2_ with different numbers of oxygen vacancies

| Samples | Vo1 | Vo2-1 | Vo2-2 | Vo2-3 | Vo3-1 | Vo3-2 | Vo3-3 | Vo3-4 |
| --- | --- | --- | --- | --- | --- | --- | --- | --- |
| Magnetic moment | 56 | 58 | 58 | 58 | 60 | 60 | 60 | 60 |
| Spin | 3.11 | 3.22 | 3.22 | 3.22 | 3.33 | 3.33 | 3.33 | 3.33 |

Table S4 Mn 2p XPS spectra analysis, O 1s XPS spectra analysis, and average oxidation state (AOS) of synthesized Mn_3_O_4_ precursors treated under different oxygen partial pressures.

| Sample | Mn 2p3/2 | | | O1s | | | AOS |
| --- | --- | --- | --- | --- | --- | --- | --- |
|  | Mn^2+^ (%) | Mn^3+^ (%) | Mn^4+^ (%) | O_latt_ (%) | O_sur_ (%) | O_ads_ (%) |  |
| 0% | 9.1 | 64.1 | 26.8 | 39.7 | 49.7 | 10.7 | 4.01 |
| 20% | 15.9 | 63.0 | 21.1 | 58.6 | 26.7 | 14.6 | 3.63 |
| 40% | 20.0 | 45.2 | 34.7 | 5.8 | 54.5 | 39.7 | 3.19 |
| 60% | 34.6 | 39.4 | 26.0 | 20.9 | 40.7 | 38.5 | 2.56 |
| 80% | 31.9 | 44.9 | 23.2 | 20.0 | 44.6 | 35.3 | 2.87 |
| 100% | 29.8 | 45.6 | 24.7 | 19.3 | 43.6 | 37.1 | 3.06 |
